# Older Adults’ Vigorous Occupational Physical Activity Levels in Six Countries Are Explained by Country and ‘Having Multiple Jobs’

**DOI:** 10.3390/ijerph192114065

**Published:** 2022-10-28

**Authors:** Nestor Asiamah, Kofi Awuviry-Newton, Edgar R. Vieira, Andrew Bateman, Hafiz T. A. Khan, Henry Kofi Mensah, Pablo Villalobos Dintrans, Emelia Danquah

**Affiliations:** 1 Division of Interdisciplinary Research and Practice, School of Health and Social Care, University of Essex, Essex, Colchester CO4 3SQ, UK; 2 Africa Centre for Epidemiology, Department of Gerontology and Geriatrics, Accra P.O. Box AN 18462, Ghana; 3 African Health and Ageing Research Centre (AHaARC), Department of Geriatrics and Gerontology, Winneba, Ghana; 4 Department of Physical Therapy, Nicole Wertheim College of Nursing & Health Sciences, Florida International University, Miami, FL 33199, USA; 5 College of Nursing, Midwifery, and Healthcare, University of West London, Paragon House, Boston Manor Road, Brentford TW8 9GB, UK; 6 Department of Human Resources and Organizational Development, Kwame Nkrumah University of Science and Technology, PMB KNUST, Kumasi, Ghana; 7 Programa Centro Salud Pública, Facultad de Ciencias Médicas, Universidad de Santiago, Santiago 8990000, Chile; 8 Millennium Institute for Care Research (MICARE), Santiago, Chile; 9 Research Directorate, Koforidua Technical University, Koforidua P.O. Box KF 981, Ghana

**Keywords:** physical activity, occupational physical activity, older adults, multiple countries

## Abstract

Several studies have compared physical activity (PA) levels between countries, but none of these studies focused on older adults and occupational PA. This study aimed to assess potential inequalities in older adults’ occupational PA across six countries and to ascertain whether having multiple jobs is a factor that interacts with country of residence to modify inequalities. This study adopted a cross-sectional design with a statistical technique screening for potential covariates. Older adults (mean age = 64 years; range = 50–114 years) from six countries (Russia, Mexico, China, India, Ghana, and South Africa) participated in the study. We utilised data from the first wave of the Study on Global AGEing and Adult Health (SAGE). These data were collected from 2007 to 2010. A random sample of 34,114 older adults completed the survey. We analysed the data with a two-way multivariate analysis of variance after screening for the ultimate covariates. There were differences in occupational PA levels (i.e., vigorous and moderate PA) among the six countries. Occupational PA levels were not significantly associated with having multiple jobs. However, having multiple jobs interacted with country of residence to influence vigorous occupational PA. Older adults from most countries who had more than one job reported more vigorous occupational PA. Older adults’ occupational PA differed among the six countries, and having multiple jobs was associated with more vigorous occupational PA. Older adults who keep multiple jobs at a time may be more active than their counterparts who had one job or were unemployed.

## 1. Introduction

Physical activity (PA) protects against long-term health conditions such as cardiovascular and neurodegenerative disorders [1,2,3,4] as well as mortality [5,6], making interventions aimed at improving PA worthwhile, especially among older adults. The implementation of PA interventions and policy requires studies comparing PA prevalence across countries [7]. Studies [5,7,8] compared PA levels across countries over the last two decades. Bauman and colleagues [8] assessed the prevalence of PA in 20 countries. Another study explored the levels of PA among schoolchildren from 34 countries [9]. Kwak et al. [7] compared occupational PA between the United States and Sweden. However, few studies assessed occupational PA prevalence. We operationally define occupational PA as physical activities performed as part of the individual’s job. No study has evaluated the level of older adults’ occupational PA with data from multiple countries.

Employment is an opportunity to keep active and perform PA, especially among men [2,7,10]. If so, PA may be directly proportional to the number of jobs an individual holds. In contrast, reduced PA is associated with jobs requiring many hours of sitting [2,7]. Therefore, having multiple inactive jobs [7] may not be associated with increased PA. This may help explain why studies have had mixed findings regarding the association between employment type (i.e., active and inactive jobs; service and manufacturing) and PA [7,10,11]. These mixed findings indicate the need to evaluate whether ‘having multiple jobs’ interacts with country of residence to modify older adults’ occupational PA levels.

The job demands–resources (JD-R) theory proposed by Demerouti et al. [12] recognises PA as a job resource for its buffering influence on job demands (e.g., stress, burnout) and its potential to benefit health and individual job performance [13]. Employers and organizations are, therefore, encouraged to roll out programmes that would increase PA as a resource against job demands including stress and burnout [13]. While we admit that this call is important, its acceptability can be enhanced with evidence regarding the relationship between occupational PA as a job resource and context or country, given that occupational PA has not been compered between countries. This comparison is important because the culture of PA among groups and organizations is affected by national PA policies and interventions [4]. Therefore, this study aims to compare occupational PA across countries for the first time, providing a basis for proffering implications for national and organizational PA policies.

Occupational PA is associated with personal characteristics, including gender, education, and age [7,11,12,14], which suggests that any differences in occupational PA explained by country of residence and multiple employment status can be dependent on these personal factors. Personal factors need to be considered as potential covariates in the association between occupational PA, country of residence, and having multiple jobs. Therefore, the objectives of this study were: (1) to assess potential inequalities in older adults’ occupational PA across six countries (i.e., Russia, Mexico, China, India, Ghana, and South Africa), (2) to ascertain whether having multiple jobs is associated with higher occupational PA, and (3) to evaluate potential interactions between having multiple jobs and country of residence on occupational PA levels. We expect this study to provide a basis for future studies comparing older adults’ occupational PA over time and across more countries.

## 2. Methods and Materials

### 2.1. Sample and Procedure

The data used in this study were from wave 1 of the World Health Organization (WHO) Study on Global AGEing and Adult Health (SAGE). This study was a cohort study performed from 2007 to 2010 on ageing and older adults from six countries, namely Russia, Mexico, China, India, Ghana, and South Africa [13,14]. The first wave of the study utilised a face-to-face individual interview to capture data in the six countries. A multistage cluster sampling was implemented by each country to determine a nationally representative cohort of older adults. Information about the study’s response rate, sampling process, and other procedures was recently published [13,14,15]. Age entries less than 50 years were removed from the data to ensure that only those aged 50 years or higher were included in our analysis. The study was approved by the WHO’s Ethics Review Board [15,16].

### 2.2. Measurement and Variable Computation

Occupational PA was measured with two domains (i.e., vigorous and moderate PA) as physical activities performed as part of the individual’s work. The two domains were measured with the WHO’s Global Physical Activity Questionnaire [16,17]. Vigorous PA was measured with two questions; one question measured weekly time (in minutes and hours) spent on work-related vigorous PA whereas the other measured the weekly number of days of vigorous PA. Moderate PA was measured with two similar questions. Table A1 shows the WHO’s formulae we used to compute vigorous and moderate-intensity PA in MET-minutes/week [2].

Having multiple jobs was measured as a categorical variable of two groups: group 1 (i.e., older adults with only one job coded as 1) and group 2 (i.e., older adults with two or more jobs coded as 2). Country of residence was created by integrating individual datasets from the six countries. The integrated data captured country as a categorical variable with the following six groups (i.e., South Africa—1; Ghana—2; India—3; Mexico—4; Russia—5, and China—6). Potential covariates included were gender, education, age, context experience, and retirement age. Gender was captured in the data as a categorical variable (i.e., men—1; women—2) whereas the other covariates were captured as continuous variables. Context experience was the number of years older adults had lived in their respective countries; retirement age was the age at which the individual stopped working for pay or income, and education was the number of years of schooling reported by the individual.

### 2.3. Statistical Analysis Procedure

We analysed data in two phases with SPSS version 28 (IBM Inc, New York, USA). In the first phase, we removed unwanted data features such as values (e.g., −8, −9) used to code uncertainty or participants’ inability to respond. We used the ‘transform variable’ function to set all such unwanted items as missing data. The analyses were then programmed to exclude missing items. The final statistical model addressing our three research questions was based on a sample of 34,114 older adults reached after removing the missing items.

The first phase includes the exploratory analysis focused on summarising the data and testing assumptions governing the use of a two-way multivariate analysis of variance (MANOVA). In this regard, descriptive statistics (i.e., frequencies for categorical variables and the mean for continuous variables) were generated on all variables. A sensitivity analysis recently used [2,18] to screen for the ultimate covariates was subsequently adopted to know if any of the covariates could affect the relationship between the two predictors and occupational PA. Since not all potential covariates can confound a relationship [18], this analysis enabled us to identify only variables likely to confound our primary relationships. Before this analysis was performed with hierarchical linear regression (HLR), all categorical variables were dummy coded since regression does not support categorical predictors. In the process, we treated occupational PA as the outcome variable and ‘having multiple jobs’ as the primary predictor [16]. The covariates were then screened with the procedure, but none of them qualified to be in the final analysis or model.

Subsequently, we assessed the following four assumptions regarding MANOVA: multivariate normality, linearity of the outcome variables, multivariate homogeneity of variances across groups, and multivariate homogeneity of covariances [19,20]. Linearity of the dependent variables was assessed by computing Pearson’s correlation between the three dependent variables (i.e., vigorous PA, moderate PA, and occupational PA). A significant correlation between these variables at *p* < 0.05 confirmed linearity [19,20]. Table A2 shows these correlations.

The remaining assumptions were assessed concurrently through the MANOVA model used to test the primary relationships of interest. Multivariate normality of the data was assessed by saving the Cook’s D values of the model and computing their corresponding probability values. The probability values indicated that multivariate normality was not achieved, but this was not a problem since the sample size was large [19,20] and the constants (i.e., 8 and 4) associated with the formulae used to compute PA (see Table A1) multiplied the variability in the data. These constants and a large sample make multivariate normality very unlikely and irrelevant. Multivariate homogeneity of variances and multivariate homogeneity of covariances were, respectively assessed with the Levene’s and Box’s M tests. Results of these tests are later presented with the main findings of this study. The above exploratory analysis provided the basis for fitting a MANOVA model, which concurrently addressed our three research questions. The statistical significance of the findings was detected at a minimum of *p* < 0.05. In accordance with previous studies [21,22], the effect sizes (i.e., partial eta squared (PES)) were interpreted as small (PES = 0.01), moderate (PES = 0.06), or large (PES = 0.14).

## 3. Results

Table 1 shows summary statistics on personal variables whereas Table 2 shows descriptive statistics on occupational PA. In Table 1, 53% (*n* = 25,180) of the participants were women whereas the average age was about 64 years (mean = 63.9; SD = 14.88; range = 50–114). In Table 2, South Africa, for example, account for a total vigorous-intensity PA of about 7088 MET-minutes/week (mean = 7088.15; SD = 7960.22). South African residents who had another job reported a higher vigorous PA (mean = 8473.85; SD = 8677.19) compared with those who did not (mean = 6892.35; SD = 7859.42). Table A3 shows Levene’s test of multivariate equality of variances, which is significant at *p* < 0.001. The footnote to Table A3 also shows the Box’s M test of the multivariate homogeneity of covariances, which is also significant at *p* < 0.001. Thus, these two assumptions were not met. Table A4 shows results of a multivariate test of the associations between PA, country of residence, and “having multiple jobs” (HMJ).

Table 2 shows the results of the multivariate test of association between occupational PA, its two domains, and the two categorical predictors. This table presents salient statistics from Table A5 in the appendix. Since the Box’s M test was significant, only the Pillai’s Trace model (in Table A4) was interpreted. For the predictor ‘Country’, the test is significant at *p* < 0.001 (F = 112.33, PES = 0.072; power = 1.0), which suggests that there is a significant difference between the six countries on occupational PA and its two domains. There was no significant difference between the two groups (in terms of ‘having multiple jobs’) on occupational PA and its two domains at *p* > 0.05 (F = 1.628; PES = 0.001; power = 0.431). Finally, there was a significant association between the interaction term (i.e., Country*HMJ) and the three outcome variables at *p* < 0.001 (F = 4.329, PES = 0.003; power = 1.0). The power corresponding to the above significant results was 1, which means that there was 100% chance that the results would have come out significant. Table 3 shows tests of between-subjects effects. Country of residence was significantly associated with vigorous-intensity PA (*p* < 0.001; PES = 0.009; power = 1.0), moderate-intensity PA (*p* < 0.001; PES = 0.034; power = 1.0), and occupational PA (*p* < 0.001; PES = 0.163; power = 1.0). These results suggest that occupational PA and its two domains differed between the six countries. Additionally, those who had one or more other jobs reported different levels of vigorous-intensity PA and occupational PA across the six countries. Regarding Table 2, older adults from four (i.e., South Africa, Ghana, India, and Russia) out of six countries who had multiple jobs reported higher vigorous-intensity PA. Older adults with multiple jobs from 3 countries (i.e., South Africa, India, and Russia) reported higher occupational PA. As seen in Table 3, the difference between countries in terms of occupational PA is strong (PES = 0.16) but is weak in terms of vigorous PA (PES = 0.01) and moderate PA (PES = 0.03) [21,22].

Table 4 shows the results of the multiple comparisons test performed concerning the relationship between country of residence and occupational PA as well as its two domains. Since we did not meet the multivariate homogeneity of variances assumption, we chose a post hoc test (i.e., Tamhane’s T2) that compensated for multivariate differences in group variances. Older adults from Ghana reported vigorous-intensity PA larger than what was reported by their counterparts from South Africa at *p* < 0.05 (see Table 2). Similarly, vigorous-intensity PA reported by Mexican older adults was larger than what was reported by South African older adults at *p* < 0.05. South Africa’s moderate-intensity PA was significantly smaller at *p* < 0.001, compared to the other five countries. Occupational PA in South Africa, though, was higher at *p* < 0.001 than what was reported for China.

## 4. Discussion

This study aimed to assess potential inequalities in older adults’ occupational PA across six countries and to ascertain whether country of residence interacts with having multiple jobs to modify these inequalities.

This study found a significant difference in older adults’ occupational PA between the countries and, thus, confirmed inequalities in occupational PA across the six countries. Inequalities between the countries were higher for moderate PA as well as occupational PA, and only South Africa reported a significantly higher vigorous-intensity PA. Our results regarding the inequalities are consistent with most studies [4,5,8]. For instance, Bauman and associates [8] reported similar inequalities in PA across 20 countries. More recently, Guthold et al. [4] reported inequalities in PA insufficiency (which reflects inequalities in PA) across a pooled analysis of 298 population-based surveys. Unlike these studies, nevertheless, our study was focused on older adults and occupational PA, which means that inequalities in PA are not limited to children [9], adolescents [5], and samples combining all age groups [4,7]. It is worth mentioning that a study [7] did not find a significant difference in occupational PA between US and Sweden, but this was based on the general population rather than on older adults. Moreover, a difference between only two countries was less likely, compared to a difference among six countries. In any case, more studies focused on older adults are needed to build a consensus regarding the association between occupational PA and country of residence.

There was no significant difference in older adults’ occupational PA between groups 1 and 2, which means that we did not find enough evidence to conclude that older adults with multiple jobs performed higher or lower occupational PA compared with their colleagues with one job. This evidence suggests that the aggregated data produced almost equal levels of PA for the two groups. Yet, having multiple jobs significantly interacted with country of residence to influence occupational PA. This result indicates that older adults with multiple jobs reported significantly different occupational and vigorous PA levels across the countries, compared with their colleagues with a single job. Similarly, the non-significant association between having multiple jobs and occupational PA was modified by country of residence, which means that those with multiple jobs reported higher vigorous-intensity PA and occupational PA for most countries (see Table 2). In the light of empirical and anecdotal evidence [2,7], we reason that older adults in group 2 who reported higher PA possibly held jobs in sectors where a significant part of work time involves vigorous PA. These sectors may include manufacturing companies demanding climbing, lifting, and other forms of manual labour. It can be said from this standpoint that opportunities for doing occupational PA (i.e., having walkable workplaces and neighbourhoods, a national PA policy, and a culture promoting PA) in most of the countries were higher among older adults with multiple jobs. In other words, differences in these opportunities between the two groups across the six countries explain the interaction between country of residence and having multiple jobs. While findings regarding this interaction make our study unique, their significance is limited without evidence on how job type (i.e., active and inactive) and sector of work (i.e., service and manufacturing) interact with having multiple jobs, country, and occupational PA. This assertion recalls some limitations of this study.

Our cross-sectional design does not establish consistency of the confirmed differences over time. For this reason, future studies employing prospective designs and examining differences between multiple countries and waves could add value to our findings. Moreover, our use of subjective measures was not necessarily free of response bias, so future researchers are encouraged to utilize objective measures such as the accelerometer or pedometer. Since the dataset used does not include a measure of the type of job [i.e., active and inactive] or employment sector (i.e., services and manufacturing), future studies including these variables and assessing their potential modification of occupational PA across countries and the two groups are highly recommended. The WHO may also consider including these measures in future waves of its PA surveillance. We also acknowledge that our data did not meet some of the assumptions governing the use of HLR analysis and MANOVA. Though this issue was owing to our relatively large sample size and constants in the formulae used to calculate PA in MET-minutes/week, the use of objective measures in future studies may be helpful. The data used in this study are old as they were collected during 2007–2010. As such, our evidence does not describe current phenomena and may not be applicable in situations where evidence from current data is needed. Yet, this study is one of several recently published studies [15,23,24,25] utilizing data of a similar age. Moreover, it has provided evidence and methods that can encourage or inform future research. Our analysis did not include older adults without a job and, therefore, does not evidence how PA may differ in this group of older adults, compared with those with one or more jobs. Finally, there were no measures for work-related walking and leisure-time PA in the SAGE.

Despite the above limitations, this study is important for some reasons. First, this study builds on research to date comparing PA across countries by focusing on older adults and occupational PA for the first time. Furthermore, this is the first study to investigate whether having multiple jobs is associated with occupational PA across multiple countries. Thus, this study sets the basis for more research investigating whether keeping multiple jobs benefits occupational PA in older adults. As mentioned earlier, though, future research would have to consider how the type of job or sector of employment modifies occupational PA across countries and between groups 1 and 2. Our effort to use HLR to screen for the ultimate covariates, rather than infusing all potential covariates into the MANOVA model, can be an example for future research. The use of a two-way MANOVA enabled us to avoid or minimise statistical bias associated with multiple independent models. Furthermore, MANOVA enabled us to answer our three research questions concurrently through a single model specification, which checked against type I error. Finally, our study serves as a foundation for similar future studies, especially those concurrently comparing occupational PA across countries, personal factors, and multiple waves. Statistics (e.g., effect size, power) reported in this study can be used for sample size calculation in future studies.

### Implications for Policy, Research, and Practice

The significant difference in occupational PA between the countries implies that older residents working in different countries can have unequal levels of occupational PA as a job resource. Given that PA protects the individual’s health, any inequalities in health and opportunities for high productivity can be attributed to the foregoing difference. This study, therefore, supports a need for interventions reducing or eliminating inequalities in occupational PA across countries. Moreover, the six countries considered in this study have national policies or programmes recognising the importance of PA [4,5]. So, the above difference in occupational PA between the countries suggests that national policies would yield unequal impacts on individual occupational PA across countries possibly due to differences in priorities and the rigor or quality of the interventions. Even if these policies accompany the same interventions and priorities across the countries, their enforcement could vary, which may have explained the differences found. Countries also adopt national PA policies at different times and would, therefore, be at different levels of policy impact and maturity. As such, countries ought to periodically assess whether their priorities and enforcement strategies are producing optimal outcomes vis à vis other countries.

The modification of the difference in occupational PA between the countries by ‘having multiple jobs’ has implications for individual, organizational, and national PA practice. Individual older adults who keep multiple jobs can maintain social and physical activities into later life, though future research is needed to know if this is possible with inactive jobs (i.e., jobs requiring sitting with screens or around a desk for most of the day). It is also worthwhile for future research to investigate whether the benefits of occupational PA from multiple jobs are buffered by job demands such as stress and burnout. Any such study will be a significant contribution to the literature since PA (as a resource) and demands (e.g., stress and burnout) can increase independently with the number of jobs held by older adults. So future evidence regarding how PA interacts with core demands (e.g., stress, burnout, and occupational sitting) to influence productivity and health outcomes would be worthwhile. For this reason, organizations should be concerned about how many other jobs their older employees keep at a time and find ways to support individuals with multiple jobs to manage stress and other demands while maximising PA from their multiple jobs. Finally, national policies and programmes should aim to reduce job demands (e.g., occupational sitting) that reduce occupational PA, especially in inactive workplaces. Aiming to reduce these demands can optimise PA and other job resources in the context of the JD-R theory.

## 5. Conclusions

Occupational PA and its two domains differed among the six countries. Therefore, we conclude that there are inequalities in occupational PA across the six countries. Though ‘having multiple jobs’ has no association with occupational PA, it interacts with country of residence to influence vigorous-intensity PA and occupational PA. Thus, older adults with multiple jobs in most of the countries reported larger vigorous-intensity PA. We conclude that older adults’ occupational PA differed between the six countries, and having multiple jobs can be associated with higher vigorous PA across the countries. An implication of our results is that organizations and countries ought to adopt policies that encourage work-related PA for employees involved in excessive occupational sitting. A key implication is that future research must investigate whether the type of employment (i.e., services and manufacturing, active and inactive) modifies occupational PA across countries as well as between group 1 (i.e., older adults with only one job) and group 2 (i.e., older adults with two or more jobs).

## Figures and Tables

**Table 1:** Summary statistics on personal variables.

Variable	Group	Frequency/Mean	Percent/SD
Country [*n* = 47,442]	South Africa	4227	9%
	Ghana	5573	12%
	India	12,198	26%
	Mexico	5448	11%
	Russia	4946	10%
	China	15,050	32%
Gender [*n* = 47,442]	Men	18,914	40%
	Women	25,180	53%
	Missing	3348	7%
Having multiple jobs [*n* = 47,442]	Group 2	4254	9%
	Group 1	29,949	63%
	Missing	13,239	28%
Age [yrs, *n* = 34,114]	---	63.90	14.88
Education [yrs, *n* = 34,114]	---	8.23	4.17
Context experience [yrs, *n* = 34,114]	---	27.54	16.77
Retirement age [yrs, *n* = 34,114]	---	48.5	20.22

Note: Results in this table were generated with descriptive statistics (i.e., frequency, percent, mean, and standard deviation). SD—standard deviation; the mean and SD apply to only continuous variables (i.e., age, education, context experience, and retirement age); the original data were used to compute summary statistics on the categorical variables in order to show the proportion of missing items. Group 1—older adults with only one job; Group 2—older adults with two or more jobs.

**Table 2:** Physical activity by country of residence and employment status [*n* = 34,114].

Country	Having Multiple Jobs	Mean	Standard Deviation
Vigorous intensity physical activity [MET-minutes/week]			
South Africa	Group 2	8473.85	8677.19
	Group 1	6892.35	7859.42
	Total	7088.15	7960.22
Ghana	Group 2	8686.98	6679.68
	Group 1	8613.21	6071.89
	Total	8628.53	6201.05
India	Group 2	8871.61	8483.91
	Group 1	6582.9	7525.13
	Total	7059.15	7788.33
Mexico	Group 2	7898.18	5500.61
	Group 1	9891.35	8306.34
	Total	9680.54	8058.78
Russia	Group 2	7778.77	7556.64
	Group 1	5980.62	7003.93
	Total	6119.6	7060.73
China	Group 2	5873.91	6084.3
	Group 1	6846.98	7861.15
	Total	6538.88	7357.21
Total	Group 2	7762.19	7380.52
	Group 1	7082.95	7272.02
	Total	7224.74	7299.52
Moderate intensity physical activity [MET-minutes/week]			
South Africa	Group 2	1924.79	3338.33
	Group 1	1465.09	3166.51
	Total	1522.01	3183.64
Ghana	Group 2	3222.72	2896.82
	Group 1	2575.5	2296.8
	Total	2709.87	2446.78
India	Group 2	4482.28	3664.15
	Group 1	4179.34	3391.98
	Total	4242.37	3451.84
Mexico	Group 2	3616.36	3515.83
	Group 1	4725.59	3759.66
	Total	4608.27	3734.04
Russia	Group 2	5043.36	3549.59
	Group 1	4765.76	3975.72
	Total	4787.22	3943.57
China	Group 2	4803.65	3760.42
	Group 1	4607.25	3841.77
	Total	4669.44	3816.1
Total	Group 2	4267.51	3582.1
	Group 1	3894.96	3493.72
	Total	3972.73	3515.36
Occupational physical activity [MET-minutes/week]			
South Africa	Group 2	297,585.4	667,767.6
	Group 1	242,330.5	328,470.5
	Total	249,171.6	384,892.8
Ghana	Group 2	10,177.82	7074.47
	Group 1	10,213.75	6448.13
	Total	10,206.29	6580.97
India	Group 2	10,486.6	8783.85
	Group 1	7897.91	7928.83
	Total	8436.59	8180.19
Mexico	Group 2	8961.68	5421.14
	Group 1	11,100.82	8330.54
	Total	10,874.57	8079.4
Russia	Group 2	9901.52	7870.97
	Group 1	7575.74	7588.78
	Total	7755.5	7632.43
China	Group 2	6989.08	6596.08
	Group 1	8023.53	8157.32
	Total	7696	7710.27
Total	Group 2	14,165.42	94,195.42
	Group 1	16,028.02	72,241.92
	Total	15,639.19	77,337.63

Note: The results in this table came from the MANOVA; this table presents descriptive statistics from this analysis. MET—metabolic equivalent; Total *n* is less than 47,442 because missing items were not included in the computation; large standard deviations were due to the constants [i.e., 8 for vigorous physical activity and 4 for moderate physical activity] in the formulae used to compute physical activity. Group 1—older adults with only one job; Group 2—older adults with two or more jobs.

**Table 3:** Tests of between-Subjects Effects [*n* = 34,114].

Source		Type III Sum of Squares	df	Mean Square	F	*p*	PES	Power
Country	VPA	3,561,756,794.409	5	712,351,358.882	13.669	<0.001	0.009	1.000
	MPA	2951,958,138.340	5	590,391,627.668	51.086	<0.001	0.034	1.000
	OPA	6091,637,907,828.640	5	1,218,327,581,565.730	280.568	<0.001	0.163	1.000
HMJ	VPA	45,932,028.488	1	45,932,028.488	0.881	0.348	0.000	0.155
	MPA	3,576,798.787	1	3,576,798.787	0.309	0.578	0.000	0.086
	OPA	19,339,813,261.002	1	1,933,9813,261.002	4.454	0.035	0.001	0.560
Country * HMJ	VPA	2,304,898,285.120	5	460,979,657.024	8.845	<0.001	0.006	1.000
	MPA	55,767,968.147	5	11,153,593.629	0.965	0.438	0.001	0.350
	OPA	69,227,330,676.863	5	13,845,466,135.373	3.188	0.007	0.002	0.889

Note: This table is one of the output tables of MANOVA; this table is only a part of a larger table. The test was significant at *p* < 0.05. VPA—vigorous intensity physical activity, MPA—moderate intensity physical activity, OPA—occupational physical activity, PES—partial Eta square; HMJ—having multiple jobs. Group 1—older adults with only one job; Group 2—older adults with two or more jobs; * Denotes interaction between country and HMJ.

**Table 4:** Post hoc and multiple comparison test (*n* = 34,114).

(I) Country	(J) Country	MD (I–J)	Std. Error	*p*	95% CI
Vigoous intensity physical activity (MET-minutes/week)					
South Africa	Ghana	−1540.37 *	527.47	0.004	±2067.99
	India	29.00	518.30	0.955	±2032.02
	Mexico	−2592.39 *	865.61	0.003	±3393.70
	Russia	968.56	545.80	0.076	±2139.85
	China	549.27	530.32	0.300	±2079.16
Ghana	South Africa	1540.3736 *	527.47	0.004	±2067.99
	India	1569.3756 *	224.76	<0.001	±881.19
	Mexico	−1052.01	728.81	0.149	±2857.37
	Russia	2508.93*	282.46	<0.001	±1107.41
	China	2089.64 *	251.24	<0.001	±985.02
India	South Africa	−29.00	518.30	0.955	±2032.02
	Ghana	−1569.38 *	224.76	<0.001	±881.19
	Mexico	−2621.39 *	722.20	<0.001	±2831.44
	Russia	939.56 *	264.93	<0.001	±1038.70
	China	520.27 *	231.36	0.025	±907.09
Mexico	South Africa	2592.39 *	865.61	0.003	±3393.70
	Ghana	1052.01	728.81	0.149	±2857.37
	India	2621.39 *	722.20	<0.001	±2831.44
	Russia	3560.94 *	742.19	<0.001	±2909.80
	China	3141.66 *	730.88	<0.001	±2865.46
Russia	South Africa	−968.56	545.80	0.076	±2139.85
	Ghana	−2508.93 *	282.46	<0.001	±1107.41
	India	−939.56 *	264.93	<0.001	±1038.70
	Mexico	−3560.94 *	742.19	<0.001	±2909.80
	China	−419.29	287.74	0.145	±1128.13
China	South Africa	−549.27	530.32	0.300	±2079.16
	Ghana	−2089.64 *	251.24	<0.001	±985.02
	India	−520.27 *	231.36	0.025	±907.09
	Mexico	−3141.66 *	730.88	<0.001	±2865.46
	Russia	419.29	287.74	0.145	±1128.13
Moderate intensity physical activity (MET-minutes/week)					
South Africa	Ghana	−1187.87 *	248.39	<0.001	±973.84
	India	−2720.37 *	244.07	<0.001	±956.90
	Mexico	−3086.26 *	407.62	<0.001	±1598.12
	Russia	−3265.21 *	257.02	<0.001	±1007.68
	China	−3147.43 *	249.73	<0.001	±979.09
Ghana	South Africa	1187.87 *	248.39	<0.001	±973.84
	India	−1532.50 *	105.84	<0.001	±414.96
	Mexico	−1898.40 *	343.20	<0.001	±1345.56
	Russia	−2077.34 *	133.01	<0.001	±521.49
	China	−1959.56 *	118.31	<0.001	±463.86
India	South Africa	2720.37 *	244.07	<0.001	±956.90
	Ghana	1532.50 *	105.84	<0.001	±414.96
	Mexico	−365.90	340.09	0.282	±1333.35
	Russia	−544.84 *	124.76	<0.001	±489.13
	China	−427.06 *	108.95	<0.001	±427.16
Mexico	South Africa	3086.26 *	407.62	<0.001	±1598.12
	Ghana	1898.40 *	343.20	<0.001	±1345.56
	India	365.90	340.09	0.282	±1333.35
	Russia	−178.95	349.50	0.609	±1370.25
	China	−61.17	344.18	0.859	±1349.37
Russia	South Africa	3265.21 *	257.02	<0.001	±1007.68
	Ghana	2077.34 *	133.01	<0.001	±521.49
	India	544.84 *	124.76	<0.001	±489.13
	Mexico	178.95	349.50	0.609	±1370.25
	China	117.78	135.50	0.385	±531.24
China	South Africa	3147.43 *	249.73	<0.001	±979.09
	Ghana	1959.56 *	118.31	<0.001	±463.86
	India	427.06 *	108.95	<0.001	±427.16
	Mexico	61.17	344.18	0.859	±1349.37
	Russia	−117.78	135.50	0.385	±531.24
Occupational physical activity (MET-minutes/week)					
South Africa	Ghana	238,965.31 *	4814.78	0.000	±18,876.77
	India	240,735.01 *	4731.04	0.000	±18,548.47
	Mexico	238,297.03 *	7901.35	<0.001	±30,977.92
	Russia	241,416.10 *	4982.10	0.000	±19,532.77
	China	241,475.60 *	4840.78	0.000	±18,978.71
Ghana	South Africa	−238,965.31 *	4814.78	0.000	±18,876.77
	India	1769.70	2051.62	0.388	±8043.54
	Mexico	−668.28	6652.64	0.920	±26,082.27
	Russia	2450.79	2578.32	0.342	±10,108.51
	China	2510.30	2293.37	0.274	±8991.35
India	South Africa	−240,735.01 *	4731.04	0.000	±18,548.47
	Ghana	−1769.70	2051.62	0.388	±8043.54
	Mexico	−2437.98	6592.29	0.712	±25,845.65
	Russia	681.09	2418.34	0.778	±9481.31
	China	740.59	2111.92	0.726	±8279.95
Mexico	South Africa	−238,297.03 *	7901.35	<0.001	±30,977.92
	Ghana	668.28	6652.64	0.920	±26,082.27
	India	2437.98	6592.29	0.712	±25,845.65
	Russia	3119.07	6774.73	0.645	±26,560.90
	China	3178.57	6671.49	0.634	±26,156.14
Russia	South Africa	−241,416.10 *	4982.10	0.000	±19,532.77
	Ghana	−2450.79	2578.32	0.342	±10,108.51
	India	−681.09	2418.34	0.778	±9481.31
	Mexico	−3119.07	6774.73	0.645	±26,560.90
	China	59.50	2626.55	0.982	±10,297.62
China	South Africa	−241,475.60 *	4840.78	0.000	±18,978.71
	Ghana	−2510.30	2293.37	0.274	±8991.35
	India	−740.59	2111.92	0.726	±8279.95
	Mexico	−3178.57	6671.49	0.634	±26,156.14
	Russia	−59.50	2626.55	0.982	±10,297.62

Note: This table came from MANOVA; * mean difference significant at *p* < 0.05; MD—mean difference; CI—confidence interval; PES—partial Eta square; HMJ—having multiple jobs; the error term is mean square [error] = 4,342,357,148.26.

## Data Availability

The data used for this study will be made available upon request.

## Appendix A

Table: Table A1, Table A2, Table A3, Table A4 and Table A5.

**Table A1:** Formulae used to compute occupational PA and its two domains.

Variable	Original Name	Formula	Note
Vigorous PA reported in hours	q3018h	---	No formula was used
Vigorous PA reported in minutes	q3018m	---	No formula was used
Moderate PA reported in hours	q3021h	---	No formula was used
Moderate PA reported in minutes	q3021m	---	No formula was used
q3018 (VPA in minutes)	---	q3018h * 60 + q3018m	q3018 is the new name assigned to VPA in minutes
q3021 (MPA in minutes)	---	q3021h * 60 + q3021m	q3021 is the new name assigned to MPA in minutes
VPA	---	8 * q3018 * q3017	q3017 is number of VPA days
MPA	---	4 * q3021 * q3020	q3020 is number of MPA days
OPA	---	VPA + MPA	

Note: * Multiplication; PA—physical activity; VPA—vigorous physical activity; MPA—moderate physical activity; OPA—occupational physical activity.

**Table A2:** Bivariate correlations between key variables of the study.

Variable	1	2	3	4	5	6	7	8	9	10	11	12	13	14
1. Vigorous physical activity	1	0.155 **	0.147 **	−0.234 **	−0.004	−.047 **	−0.095 **	−0.046 *	−0.033 **	−0.005	0.182 **	−0.135**	0.092 **	−0.061 **
2. Moderate physical activity		1	−0.012	0.020 **	−0.165 **	−0.040 **	−0.071 **	−0.067 **	−0.063 **	−0.205 **	−0.013	0.097 **	0.053 **	0.032 **
3. Occupational physical activity			1	−0.019	0.031 **	−0.003	−0.040 *	−0.102 **	0.019	0.497 **	−0.048 **	−0.115 **	−0.011	−0.049 **
4. Gender				1	−0.094 **	−0.044 **	0.086 **	−0.100 **	0.085 **	−0.006	−0.080 **	0.049 **	0.021 **	0.049 **
5. Age (yrs)					1	−0.147 **	0.480 **	0.286 **	0.130 **	0.080 **	0.090 **	−0.343 **	0.122 **	0.133 **
6. Education (yrs)						1	−0.115 **	0.056 **	0.057 **	−0.038 **	0.063 **	−0.081 **	−0.179 **	0.333 **
7. Context experience (yrs)							1	0.137 **	0.022 *	−0.122 **	−0.108 **	0.069 **	0.099 **	0.141 **
8. Retirement age (yrs)								1	0.008	−0.147 **	−0.094 **	−0.009	−0.039 **	0.153 **
9. Having multiple jobs									1	0.079 **	−0.093 **	−0.048 **	0.028 **	0.084 **
10. South Africa										1	−0.153 **	−0.255 **	−0.151 **	−0.143 **
11. Ghana											1	−0.299 **	−0.177 **	−0.167 **
12. India												1	−0.295 **	−0.279 **
13. Mexico													1	−0.165 **
14. Russia														1

** *p* < 0.001; * *p* < 0.05. The group ‘men’ set as reference for gender; Group 2 set as reference for ‘having multiple jobs’, and ‘China’ set as reference for country of residence.

**Table A3:** Levene’s test of equality of error variances.

Domain	Note	Statistic	df1	df2	*p*
Vigorous intensity PA	Based on mean	14.429	11	7207	<0.001
	Based on median	7.303	11	7207	<0.001
	Based on Median and with adjusted df	7.303	11	6681.903	<0.001
	Based on trimmed mean	11.62	11	7207	<0.001
Moderate intensity PA	Based on mean	42.906	11	7207	<0.001
	Based on median	34.112	11	7207	<0.001
	Based on Median and with adjusted df	34.112	11	6786.429	<0.001
	Based on trimmed mean	41.628	11	7207	<0.001
Occupational physical activity	Based on mean	461.964	11	7207	<0.001
	Based on median	211.502	11	7207	<0.001
	Based on Median and with adjusted df	211.502	11	114.394	<0.001
	Based on trimmed mean	324.739	11	7207	<0.001

Note: Box’s M = 52,624.79; F = 782.16; df1 = 66; df2 = 45,420.08; *p* < 0.001.

**Table A4:** A multivariate test of the associations between occupational PA, country of residence, and HMJ.

Model	Value	F	Hypothesis df	Error df	*p*	PES	Power
Intercept							
Pillai’s Trace **	0.219	674.644	3	7205	<0.001	0.219	1.000
Wilks’ Lambda	0.781	674.644	3	7205	<0.001	0.219	1.000
Hotelling’s Trace	0.281	674.644	3	7205	<0.001	0.219	1.000
Roy’s Largest Root	0.281	674.644	3	7205	<0.001	0.219	1.000
Country							
Pillai’s Trace **	0.217	112.333	15	21,621	<0.001	0.072	1.000
Wilks’ Lambda	0.79	117.918	15	19,890.23	<0.001	0.075	1.000
Hotelling’s Trace	0.256	122.884	15	21,611	<0.001	0.079	1.000
Roy’s Largest Root	0.213	306.614	5	7207	<0.001	0.175	1.000
HMJ							
Pillai’s Trace **	0.001	1.628	3	7205	0.181	0.001	0.431
Wilks’ Lambda	0.999	1.628	3	7205	0.181	0.001	0.431
Hotelling’s Trace	0.001	1.628	3	7205	0.181	0.001	0.431
Roy’s Largest Root	0.001	1.628	3	7205	0.181	0.001	0.431
Country * HMJ							
Pillai’s Trace **	0.009	4.329	15	21,621	<0.001	0.003	1.000
Wilks’ Lambda	0.991	4.332	15	19,890.23	<0.001	0.003	1.000
Hotelling’s Trace	0.009	4.335	15	21,611	<0.001	0.003	1.000

* Denotes interaction between country and HMJ; ** Model interpreted; HMJ—having multiple jobs; PA—physical activity; PES—partial Eta square.

**Table A5:** Tests of between-subjects effects.

Source		Type III Sum of Squares	df	Mean Square	F	*p*	PES	Power ^d^
Corrected Model	VPA	9,000,035,101.970 ^a^	11	818,185,009.270	15.699	<0.001	0.023	1.000
	MPA	5,907,924,127.474 ^b^	11	537,084,011.589	46.473	<0.001	0.066	1.000
	OPA	11,876,277,770,765.406 ^c^	11	1,079,661,615,524.130	248.635	<0.001	0.275	1.000
Intercept	VPA	50,882,887,460.512	1	50,882,887,460.512	976.349	<0.001	0.119	1.000
	MPA	12,292,797,117.126	1	12,292,797,117.126	1063.677	<0.001	0.129	1.000
	OPA	2,375,255,266,692.880	1	2,375,255,266,692.880	546.997	<0.001	0.071	1.000
Country	VPA	3,561,756,794.409	5	712,351,358.882	13.669	<0.001	0.009	1.000
	MPA	2,951,958,138.340	5	590,391,627.668	51.086	<0.001	0.034	1.000
	OPA	6,091,637,907,828.640	5	1,218,327,581,565.730	280.568	<0.001	0.163	1.000
HAJ	VPA	45,932,028.488	1	45,932,028.488	0.881	0.348	0.000	0.155
	MPA	3,576,798.787	1	3,576,798.787	0.309	0.578	0.000	0.086
	OPA	19,339,813,261.002	1	1,933,981,3261.002	4.454	0.035	0.001	0.560
Country * HAJ	VPA	2,304,898,285.120	5	460,979,657.024	8.845	<0.001	0.006	1.000
	MPA	55,767,968.147	5	11,153,593.629	0.965	0.438	0.001	0.350
	OPA	69,227,330,676.863	5	13,845,466,135.373	3.188	0.007	0.002	0.889
Error	VPA	375,596,083,594.421	7207	52,115,454.918				
	MPA	83,290,520,114.825	7207	11,556,891.927				
	OPA	31,295,367,967,471.600	7207	4,342,357,148.255				
Total	VPA	761,405,684,096.000	7219					
	MPA	203,133,135,165.750	7219					
	OPA	44,937,300,502,075.700	7219					
Corrected Total	VPA	384,596,118,696.391	7218					
	MPA	89,198,444,242.299	7218					
	OPA	43,171,645,738,237.000	7218					

Note: * Denotes interaction between country and HMJ; VPA—vigorous intensity physical activity, MPA—moderate intensity physical activity, OPA—occupational physical activity, PES—partial Eta square; HAJ—having multiple jobs. a. R Squared = 0.023 (Adjusted R Squared = 0.022). b. R Squared = 0.066 (Adjusted R Squared = 0.065). c. R Squared = 0.275 (Adjusted R Squared = 0.274). d. Computed using alpha = 0.05.

## References

[1] Asiamah N. (2017). Social engagement and physical activity: Commentary on why the activity and disengagement theories of ageing may both be valid. Cogent Med..

[2] Asiamah N., Vieira E.R., Kouveliotis K., Gasana J., Awuviry-Newton K., Eduafo R. (2021). Associations between older African academics’ physical activity, walkability and mental health: A social distancing perspective. Health Promot. Int..

[3] Hallal P.C., Andersen L.B., Bull F.C., Guthold R., Haskell W., Ekelund U., Lancet Physical Activity Series Working Group (2012). Global physical activity levels: Surveillance progress, pitfalls, and prospects. Lancet.

[4] Guthold R., Stevens G.A., Riley L.M., Bull F.C. (2018). Worldwide trends in insufficient physical activity from 2001 to 2016: A pooled analysis of 358 population-based surveys with 1.9 million participants. Lancet Glob. Health.

[5] Guthold R., Stevens G.A., Riley L.M., Bull F.C. (2020). Global trends in insufficient physical activity among adolescents: A pooled analysis of 298 population-based surveys with 1.6 million participants. Lancet Child Adolesc. Health.

[6] Chen P.W., Chen L.K., Huang H.K., Loh C.H. (2022). Productive Aging by Environmental Volunteerism: A Systematic Review. Arch. Gerontol. Geriatr..

[7] Kwak L., Berrigan D., Van Domelen D., Sjöström M., Hagströmer M. (2016). Examining differences in physical activity levels by employment status and/or job activity level: Gender-specific comparisons between the United States and Sweden. J. Sci. Med. Sport.

[8] Bauman A., Bull F., Chey T., Craig C.L., Ainsworth B.E., Sallis J.F., Bowles H.R., Hagstromer M., Sjostrom M., Pratt M. (2009). The International Prevalence Study on Physical Activity: Results from 20 countries. Int. J. Behav. Nutr. Phys. Act..

[9] Guthold R., Cowan M.J., Autenrieth C.S., Kann L., Riley L.M. (2010). Physical Activity and Sedentary Behavior Among Schoolchildren: A 34-Country Comparison. J. Pediatr..

[10] Csizmadi I., Lo Siou G., Friedenreich C.M., Owen N., Robson P.J. (2011). Hours spent and energy expended in physical activity domains: Results from The Tomorrow Project cohort in Alberta, Canada. Int. J. Behav. Nutr. Phys. Act..

[11] Asiamah N. (2016). Socio-demographic determinants of physical activity (PA): A working class perspective. Cogent Med..

[12] Demerouti E., Bakker A.B., Nachreiner F., Schaufeli W.B. (2001). The job demands-resources model of burnout. J. Appl. Psychol..

[13] Fodor D.P., Pohrt A., Gekeler B.S., Knoll N., Heuse S. (2020). Intensity matters: The role of physical activity in the job demands-resources model. J. Work Organ. Phychol..

[14] Pan S.Y., Cameron C., DesMeules M., Morrison H., Craig C.L., Jiang X. (2009). Individual, social, environmental, and physical environmental correlates with physical activity among Canadians: A cross-sectional study. BMC Public Health.

[15] Ruan Y., Guo Y., Zheng Y., Huang Z., Sun S., Kowal P., Shi Y., Wu F. (2018). Cardiovascular disease (CVD) and associated risk factors among older adults in six low-and middle-income countries: Results from SAGE Wave 1. BMC Public Health.

[16] Kowal P., Chatterji S., Naidoo N., Biritwum R., Fan W., Lopez Ridaura R., Maximova T., Arokiasamy P., Phaswana-Mafuya N., Williams S. (2012). Data Resource Profile: The World Health Organization Study on global AGEing and adult health (SAGE). Int. J. Epidemiol..

[17] Awuviry-Newton K., Amoah D., Tavener M., Afram A.A., Dintrans P.V., Byles J., Kowal P. (2022). Food Insecurity and Functional Disability Among Older Adults in Ghana: The Role of Sex and Physical Activity. J. Am. Med. Dir. Assoc..

[18] Bempong A.E., Asiamah N. (2022). Neighbourhood walkability as a moderator of the associations between older Ghanaians’ social activity, and the frequency of walking for transportation: A cross-sectional study with sensitivity analyses. Arch. Gerontol. Geriatr..

[19] Bathke A.C., Friedrich S., Pauly M., Konietschke F., Staffen W., Strobl N., Holler Y. (2018). Testing Mean Differences among Groups: Multivariate and Repeated Measures Analysis with Minimal Assumptions. Multivar. Behav. Res..

[20] Garson G.D. (2012). Testing Statistical Assumptions.

[21] Pautz N., Olivier B., Steyn F. (2018). The use of parametric effect sizes in single study musculoskeletal physiotherapy research: A practical primer. Phys. Ther. Sport.

[22] Tsigilis N. (2006). Can secondary school students’ self-reported measures of height and weight be trusted? An effect size approach. Eur. J. Public Health.

[23] Conn A.N.D., Chan K., Banks J., Ruppar T.M., Scharff J. (2014). Cultural relevance of physical activity intervention research with underrepresented populations. Int. Quat. Commun. Health Educ..

[24] Awuviry-Newton K., Wales K., Tavener M., Kowal P., Byles J. (2021). Functional difficulties and toileting among older adults in Ghana: Evidence from the World Health Organization Study on global AGEing and adult health (SAGE) Ghana Wave 1. Ageing Soc..

[25] Larnyo E., Dai B., Nutakor J.A., Ampon-Wireko S., Larnyo A., Appiah R. (2022). Examining the impact of socioeconomic status, demographic characteristics, lifestyle and other risk factors on adults’ cognitive functioning in developing countries: An analysis of five selected WHO SAGE Wave 1 Countries. Int. J. Equity Health.

